# A CT-Based Radiomics Nomogram for Preoperative Prediction of Lymph Node Metastasis in Periampullary Carcinomas

**DOI:** 10.3389/fonc.2021.632176

**Published:** 2021-07-29

**Authors:** Lei Bi, Yubo Liu, Jingxu Xu, Ximing Wang, Tong Zhang, Kaiguo Li, Mingguang Duan, Chencui Huang, Xiangjiao Meng, Zhaoqin Huang

**Affiliations:** ^1^Department of Radiology, Shandong Provincial Hospital Affiliated to Shandong First Medical University, Jinan, China; ^2^Department of Radiology, Linyi People’s Hospital, Linyi, China; ^3^Department of Research Collaboration, R&D Center, Beijing Deepwise & League of PHD Technology Co., Ltd., Beijing, China; ^4^Department of Radiation Oncology, Shandong Cancer Hospital and Institute, Shandong First Medical University and Shandong Academy of Medical Sciences, Jinan, China

**Keywords:** periampullary carcinoma, computed tomography, radiomics, nomogram, lymph node metastasis

## Abstract

**Purpose:**

To establish and validate a radiomics nomogram for preoperatively predicting lymph node (LN) metastasis in periampullary carcinomas.

**Materials and Methods:**

A total of 122 patients with periampullary carcinoma were assigned into a training set (n = 85) and a validation set (n = 37). The preoperative CT radiomics of all patients were retrospectively assessed and the radiomic features were extracted from portal venous-phase images. The one-way analysis of variance test and the least absolute shrinkage and selection operator regression were used for feature selection. A radiomics signature was constructed with logistic regression algorithm, and the radiomics score was calculated. Multivariate logistic regression model integrating independent risk factors was adopted to develop a radiomics nomogram. The performance of the radiomics nomogram was assessed by its calibration, discrimination, and clinical utility with independent validation.

**Results:**

The radiomics signature, constructed by seven selected features, was closely related to LN metastasis in the training set (p < 0.001) and validation set (p = 0.017). The radiomics nomogram that incorporated radiomics signature and CT-reported LN status demonstrated favorable calibration and discrimination in the training set [area under the curve (AUC), 0.853] and validation set (AUC, 0.853). The decision curve indicated the clinical utility of our nomogram.

**Conclusion:**

Our CT-based radiomics nomogram, incorporating radiomics signature and CT-reported LN status, could be an individualized and non-invasive tool for preoperative prediction of LN metastasis in periampullary carcinomas, which might assist clinical decision making.

## Introduction

Periampullary carcinomas (PCs) arise within 2 cm of the major duodenal papilla, including a heterogeneous group of malignant tumors originating from the pancreas, distal common bile duct (CBD), duodenum, and ampulla of Vater. Although rare, accounting for roughly 0.2% of gastrointestinal tumors (1–3), PC is one of the top five leading causes of cancer-related death worldwide (4, 5), and the detection rate has substantially increased in the past few years (6). Traditionally, the treatment protocol is curative pancreaticoduodenectomy for resectable PCs, but the prognosis is still poor, with 5-year survival rates ranging from 6.5 to 32.8% after surgery (7). Even if the tumor was completely resected, majority of the PC patients still experience local recurrence and/or distant metastases, resulting in the low curative rate of PC (8).

PC is associated with a high rate of lymph node metastasis (LNM), which has made a considerable proportion of tumors unresectable, and has been considered to be one of the strongest predictors for patient survival (7, 9). Recent studies reported that patients with LN metastasis could obtain a survival benefit from neoadjuvant chemotherapy (10, 11). Therefore, accurate preoperative assessment of LN metastasis is essential for treatment strategy decisions and could help predict prognosis of patients with PC. However, preoperative diagnosis of LN metastasis remains challenging.

CT imaging is normally chosen for preoperative diagnosis of PCs. It provides precise anatomy with favorable resolution and can accurately predict the resectability of PCs (12); however, its ability to detect LN metastasis is unsatisfactory, with diagnostic accuracy varying from 63 to 81% (13). Correlation between intratumor heterogeneity and metastasis has been proposed (14), but information obtained from conventional CT images is limited to some simple factors (such as tumor size, shape, density, and enhancement pattern) and appears insufficient for more in-depth research.

Radiomics is a recently developed omics-based approach applied to quantitative radiology to extract multiple quantitative features from original images (15). Through automatic feature extraction algorithm, imaging data are converted into high-dimensional feature data, which have been revealed to have an underlying relationship with pathophysiological characteristics and could be used to develop predictive models to improve diagnostic and prognostic accuracy (16). Radiomics nomograms, which are constructed by incorporating quantitative risk factors, have been widely accepted as a reliable tool for predicting clinical events and have successfully assisted preoperative prediction of LN metastasis in several types of malignant tumors (17–21). Till now, there has been no published research that has evaluated whether CT-based radiomics would facilitate LNM prediction in PCs.

Therefore, in this study, we sought to establish and validate a radiomics nomogram that would incorporate a radiomics signature and clinical risk factors for preoperatively predicting LNM in PCs.

## Materials and Methods

### Study Population

**Figure 1:**
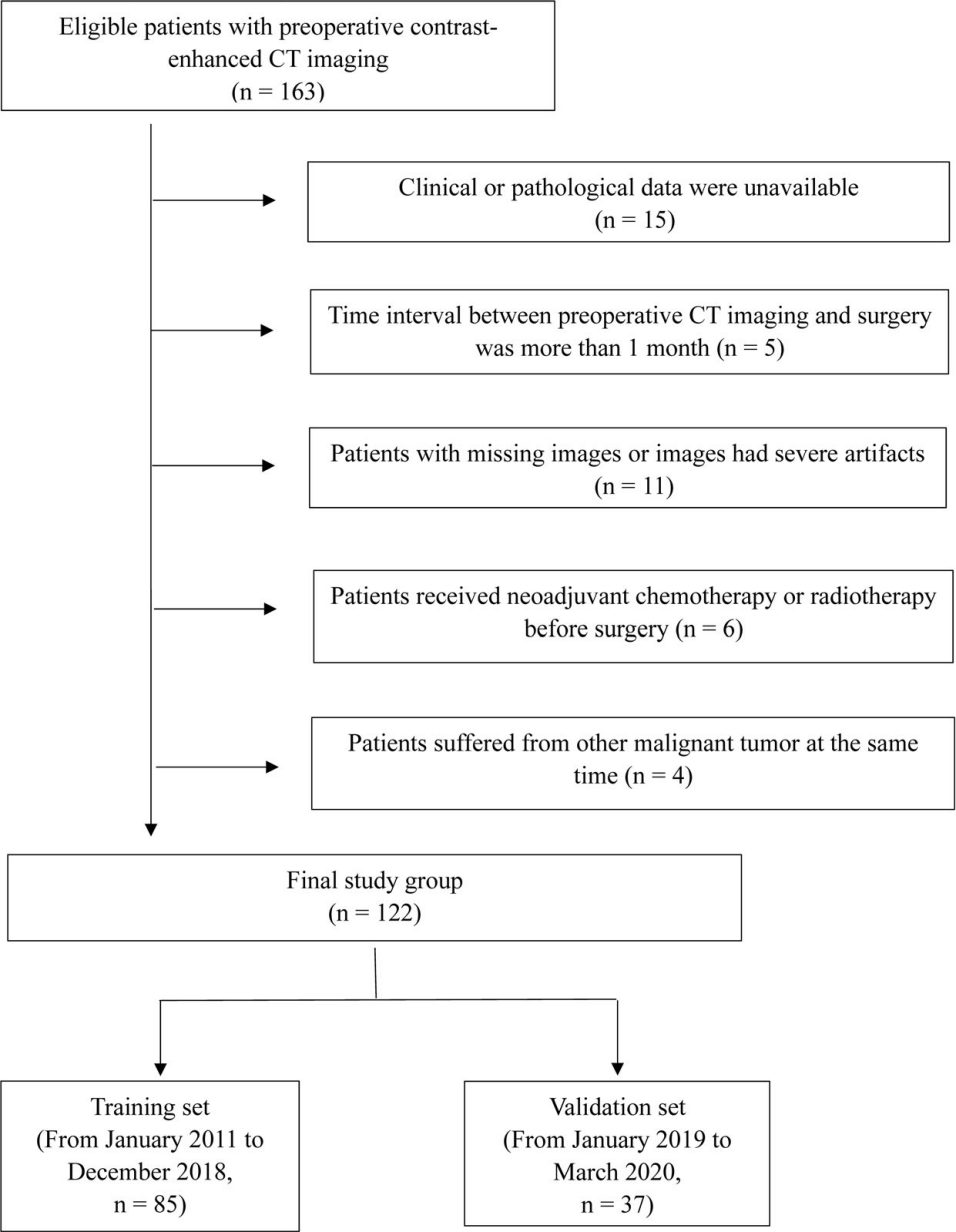
Flow diagram of patient selection procedure.

This retrospective study of anonymous data was approved by the Ethics Committee of our institution, and requirement for informed consent was waived. Preoperative CT images of patients with pathologically confirmed PC were collected from our database. The inclusion criteria were as follows: 1) patients were treated with curative-intent tumor resection and LN dissection; 2) clinical and pathological data were available; 3) maximum diameter of the tumor was larger than 3 mm; 4) preoperative CT examination was performed less than two weeks before surgery; and 5) CT images had high quality (without artifacts) for segmentation. The exclusion criteria were as follows: 1) patients have received chemotherapy or radiotherapy prior to surgery; 2) patients suffered from other malignant tumors at the same time. From January 2011 to March 2020, a total of 122 patients (mean age, 58.6 years; range, 32–78 years) were included in our study. Based on the time of surgery, patients were divided into two independent sets: 85 patients treated between January 2011 and December 2018 were included in the training set, whereas 37 patients treated between January 2019 and March 2020 were assigned into the validation set (**Figure 1**).

Clinical data including age, gender, tumor origin (pancreas, CBD, duodenum, or ampulla of Vater), preoperative serum carbohydrate antigen 19-9 (CA19-9), carbohydrate antigen 125 (CA125), carcinoembryonic antigen (CEA) levels, and dates of preoperative CT examination were obtained from our medical database. The threshold values for the levels of CA 19-9 level, CA 125, and CEA were defined as 39 U/ml, 39 U/ml, and 10 ng/ml, respectively, and values greater than those were considered to be abnormal in our institution.

### CT Image Acquisition

CT examinations were performed with 640-slice MDCT (Aquilion ONE, Toshiba, Japan) or 64-slice MDCT (CT750 HD, GE Healthcare, Wisconsin, USA) systems. Patients were required to fast for more than 4 h before examination. The tube voltage was 120 kV, the tube current was automatic milliamps, the layer thickness was 5 mm, and the reconstruction layer interval was 5 mm. After plain scanning, arterial phase (25–30 s), portal venous phase (60–70 s), and delayed phase (3 min) scans were performed. With a power injector, contrast agent (Iohexol, 300 mg iodine/ml, Beijing Beilu Pharmaceutical Co., Ltd.) was administered intravenously at a rate of 3 ml/s for a total dose of 80–85 ml, followed by a 20-ml saline flush. Furthermore, patients took 500–1,000 ml of pure water orally as a negative contrast agent before image acquisition.

### CT Feature Evaluation

Two radiologists (reader 1 and reader 2, with 7 and 14 years of experience in abdominal CT interpretation, respectively) reviewed all images in consensus, and the following traits were evaluated: 1) tumor size, defined as the maximum diameter on transverse images; 2) vascular involvement, defined as vessel occlusion, stenosis, or contour deformity associated with tumor invasion (17, 22); and 3) LN metastasis, defined as short-axis diameter larger than 1.0 cm, with central necrosis, or hyperenhanced than liver parenchyma in portal venous phase (17, 22). The observers knew that all patients received diagnoses of PC but were blind to the clinical or pathologic details.

### Tumor Segmentation and Radiomic Feature Extraction

**Figure 2:**
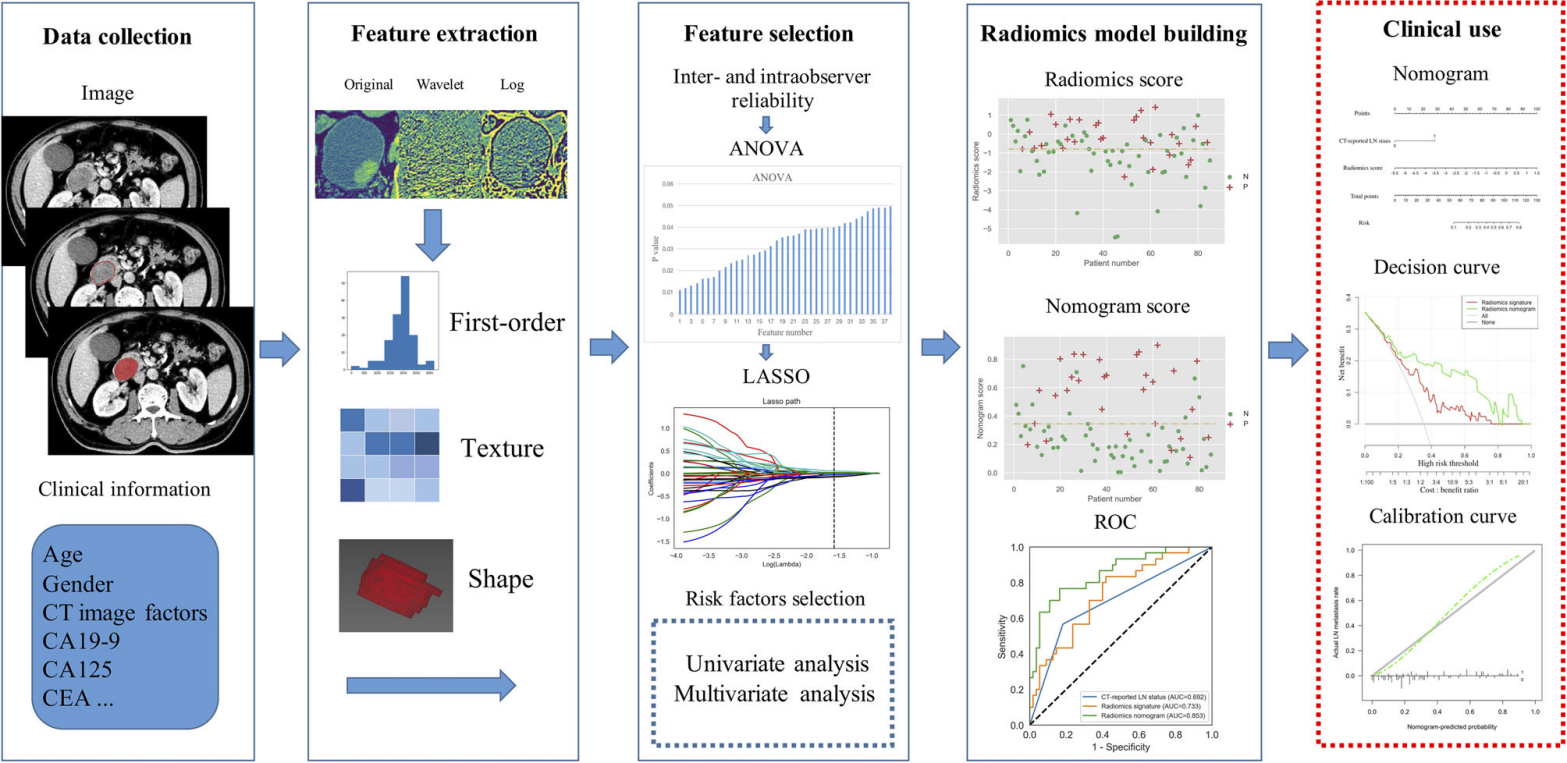
Flow diagram of radiomics procedure.

Three-dimensional (3-D) segmentation and feature extraction were performed using a postprocessing platform (Dr. Wise Multimodal Research Platform, Beijing Deepwise & League of PHD Technology Co., Ltd, Beijing, China. https://research.deepwise.com). On the portal venous-phase images, the region of interest (ROI) was manually drawn freehand on each transverse section strictly within the border of the lesion layer by layer, which should include cyst, hematoma, or necrosis within the lesion, but avoid CBD, main pancreatic duct, and other normal anatomical structures. The volume of interest (VOI) of each lesion was then automatically generated (**Figure 2**). A total of 1,218 features, including 252 first-order features, 14 shape features, and 952 texture features, were extracted from the VOI of each patient.

### LN Status-Related Feature Selection and Signature Construction

To decrease the impact from the different value scales of the radiomics features, all features were normalized before feature selection (18). Each feature was subtracted by the mean value of the training set and was divided by the standard deviation value of this set. The same normalization approach was then applied to the validation set by utilizing the mean value and standard deviation value calculated from the training set (18).

We devised a three-step procedure to reduce feature dimensionality and select the most robust and LN status-related features. First, we tested feature robustness and reproducibility by using inter- and intraclass correlation coefficients (ICCs). Twenty patients were randomly selected from the entire patient group for this analysis. To assess interobserver reliability, reader 1 and reader 2 performed the segmentation of these 20 tumors independently. To evaluate intraobserver reproducibility, reader 1 repeated the segmentation procedure after two weeks. The remaining image segmentation was also completed by reader 1. Radiomic features with both kinds of ICC values >0.90 were considered to be robust and stable and were selected for subsequent analysis. Second, we used one-way analysis of variance (ANOVA) to select features that were significantly different between LNM-positive group and LNM-negative group. Finally, to avoid the “curse of dimensionality”, which would lead to a large false positive result (23), the least absolute shrinkage and selection operator (LASSO) feature selection algorithm was applied to screen the most informative image features extracted from all VOIs, with penalty parameter tuning conducted by five-fold cross-validation. During the feature selection process, most of the covariate coefficients were reduced to zero, and the variables that still had a non-zero coefficient after the shrinking process were selected. A radiomic signature was generated through a linear combination of selected features weighted by their respective coefficients (24). The predictive accuracy of the radiomics signature was quantified by the area under the receiver operating characteristic (ROC) curve (AUC) in both training and validation sets.

### Development, Performance, and Validation of a Radiomics Nomogram

The radiomics signature and clinical factors were tested in a multivariate logistic regression model for predicting LN metastasis in the training set. Clinical factors included age, gender, tumor origin, preoperative serum CA19-9, CA125, CEA levels, and CT-reported LN status. Collinearity diagnosis was performed to detect the multicollinearity among the variables by calculating the variance inflation factor (VIF). Variables with VIF >10 indicated severe multicollinearity (25). A radiomics nomogram was subsequently established by using the selected significant covariates based on the proposed multivariate model, and the nomogram score was calculated.

The discriminative efficacy of the nomogram was quantified by ROC curve and AUC value. Calibration curves, a key measurement of the consistency between predicted LNM probability and actual LNM rate, were generated to evaluate the calibration of the nomogram, accompanied by Hosmer–Lemeshow (H–L) test to assess the goodness-of-fit of the nomogram (18). The performance of the nomogram was subsequently tested internally in an independent validation set by using the formula derived from the training set. Calibration and the H–L test were performed, and the AUC was calculated.

### Clinical Utility of the Radiomics Nomogram

Decision curve analysis (DCA) was performed to estimate the clinical utility of the radiomics signature and radiomics nomogram by calculating the net benefits for a range of threshold probabilities in the training and validation sets (24). Flow chart of the radiomics procedure was presented in **Figure 2**.

### Pathological Diagnosis of LNM

All the patients underwent lymphadenectomy, and the resected LN areas included peripancreatic, retropancreatic, hepatoduodenal, celiac, superior mesenteric, portocaval, and aortocaval (26). The resected LNs of each area were grouped and sent for histopathological examination. Each LN was subsequently dissected by the pathologist for diagnosis of LN status. LNM means that at least one of the LNs was positive.

### Statistical Analysis

Univariate analysis was applied to compare the differences in the clinical and radiomics factors between LNM-positive group and LNM-negative group. Categorical variables were compared by using the Chi-square test or Fisher exact test, and continuous variables were compared by using the Student’s *t*-test or Mann–Whitney *U* test, as appropriate. One-way ANOVA was used to compare the value of each radiomics feature between two groups. The correlation between radiomics features and the LN status was assessed by using the Kendall’s rank coefficient test. Differences in the AUC values between different models were compared using DeLong’s test. Statistical analyses were performed using SPSS software (SPSS for Windows, v. 17.0). A two-sided p value <0.05 was considered statistically significant.

## Results

### Patient Characteristics

The rates of LNM were 35.29% (30 of 85) in the training set and 35.14% (13 of 37) in the validation set, and there was no significant difference between the two sets (p = 0.98). In total, 19 patients (44.19%; 19 of 43) with LNM were understaged, and 15 patients (18.99%; 15 of 79) without LNM were overstaged based on CT-reported LN status. The overall accuracy of CT-reported LN status was 72.13% (88 of 122), with a sensitivity of 55.81% (24 of 43), a specificity of 81.01% (64 of 79), a positive predictive value (PPV) of 61.54% (24 of 39), and a negative predictive value (NPV) of 77.11% (64 of 83). No significant differences in other clinical and radiological factors between the training and validation sets were observed (p > 0.05). The baseline characteristics of all patients in the training and validation sets are listed in **Tables 1** and **S1**.

**Table 1:** Characteristics of all patients in the training set and validation set.

Characteristic	Training set (n = 85)			Validation set (n = 37)		
	Negative for LN status	Positive for LN status	P-value	Negative for LN status	Positive for LN status	P-value
Age (mean ± SD)	57.35 ± 12.18	58.40 ± 11.54	0.694	62.38 ± 10.69	57.00 ± 10.10	0.142
Gender			0.872			0.872
Male	16 (29.1)	9 (30.0)		10 (41.7)	5 (38.5)	
Female	39 (70.9)	21 (70.0)		14 (58.3)	8 (61.5)	
Tumor size (>3 cm)	6 (10.9)	10 (33.3)	0.025	4 (16.7)	3 (28.1)	0.972
CT-reported LN status			<0.001			0.093
LN negative	45 (81.8)	13 (43.3)		19 (79.2)	6 (46.2)	
LN positive	10 (18.2)	17 (56.7)		5 (20.8)	7 (53.8)	
CT-reported vascular invasion	15 (27.3)	15 (50.0)	0.063	7 (29.2)	6 (46.2)	0.501
Tumor origin			0.010			0.397
Duodenum	21 (38.2)	3 (10.0)		6 (25.0)	4 (30.7)	
Ampulla of Vater	10 (18.2)	6 (20.0)		6 (25.0)	2 (15.4)	
Common bile duct	9 (16.3)	3 (10.0)		6 (25.0)	1 (7.7)	
Pancreas	15 (27.3)	18 (60.0)		6 (25.0)	6 (46.2)	
CA 19-9 (>39 U/ml)	41 (74.6)	22 (73.3)	0.891	16 (66.7)	10 (76.9)	0.783
CA 125 (>39 U/ml)	1 (1.8)	2 (6.7)	0.587	2 (8.3)	0 (0)	0.758
CEA (>10 ng/ml)	2 (3.6)	2 (6.7)	0.925	2 (8.3)	2 (15.4)	0.916
Radiomics score (mean ± SD)	−1.19 ± 1.41	−0.17 ± 0.93	<0.001	−1.23 ± 1.01	−0.47 ± 0.81	0.017

Data are number of patients; data in parentheses are percentage unless otherwise indicated. CA 125, carbohydrate antigen 125; CA 19-9, carbohydrate antigen 19-9; CEA, carcinoembryonic antigen; LN, lymph node; SD, standard deviation.

### LN Status-Related Feature Selection and Radiomics Signature Construction

**Figure 3:**
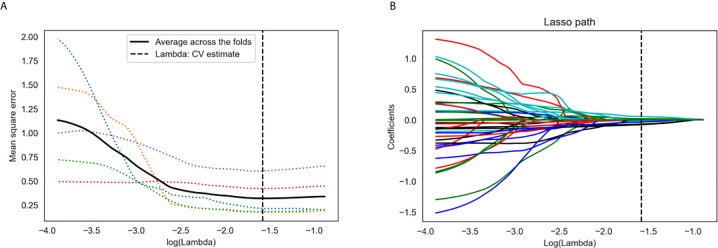
Radiomics feature selection using the least absolute shrinkage and selection operator (LASSO) regression algorithm. **(A)** Tuning parameter (*λ*) selection in LASSO model used five-fold cross-validation *via* minimum criteria. The y-axis indicates the mean square error. The x-axis indicates the log(*λ*). The black curve indicates average error for each model with given *λ*. The vertical lines define the optimal *λ* value of 0.026 with log(*λ*) = −1.579 was chosen. **(B)** LASSO coefficient profiles of the 38 radiomics features. A coefficient profile plot was generated *versus* the selected log(*λ*) value using five-fold cross-validation. A vertical line was plotted at the optimal *λ* value, which resulted in seven features with non-zero coefficients.

A total of 767 stable features (14 shape features, 162 first-order features, and 591 texture features) were selected after inter- and intraobserver reproducibility analysis. Among them, 38 radiomics features showing significant differences between two groups (p = 0.01–0.05) by one-way ANOVA were enrolled into the LASSO regression algorithm to select most LN status-related features (**Figure 3**). Finally, seven features with non-zero coefficients in LASSO regression algorithm were selected based on the training set and were used to build the logistic regression (LR) model. Among the seven features, four features were significantly correlated with the actual LN status in the Kendall’s rank correlation test, and six features were significantly different between two groups in the Mann–Whitney *U* test. The details are listed in **Table 2**.

**Table 2:** Univariate analysis and correlation test for radiomics features used in the LR model for the training set.

Radiomics features	Training set (n = 85)		P-value of univariate analysis	Correlation coefficient	P-value of correlation test
	Negative for LN metastasis	Positive for LN metastasis			
wavelet-LLH_glcm_ClusterShade	−0.078 (−0.218, 0.062)	0.053 (−0.000, 0.106)	0.075	−0.131	0.149
wavelet-HHH_glszm_SmallAreaEmphasis	0.044 (0.009, 0.079)	0.117 (0.073, 0.162)	0.018	−0.189	0.035
wavelet-HHL_glcm_Imc2	0.190 (0.167, 0.213)	0.239 (0.211, 0.268)	0.029	−0.170	0.058
original_glszm_LowGrayLevelZoneEmphasis	0.237 (0.194, 0.279)	0.336 (0.286, 0.386)	0.017	−0.190	0.034
original_glszm_SmallAreaLowGrayLevelEmphasis	0.086 (0.062, 0.110)	0.139 (0.110, 0.169)	0.016	−0.192	0.032
wavelet-LHH_glcm_SumSquares	0.249 (0.2492, 0.2497)	0.246 (0.243, 0.248)	0.036	0.162	0.071
wavelet-LLH_glszm_GrayLevelNonUniformity	4.540 (3.397, 5.683)	3.397 (2.213, 3.371)	0.005	0.244	0.009

LN, lymph node; LR, logistic regression. The univariate analysis for radiomics features was applied by using the Mann–Whitney U test. The correlation between radiomics features and the LN status was applied by using the Kendall’s rank correlation test. All features were reported as median and 95% confidence interval.

The radiomics signature was constructed, and the radiomics score was calculated by using the following formula: Radiomics score = −0.81645684 + 0.279262 * wavelet-LLH_glszm_GrayLevelNonUniformity − 0.08749282 * original_glszm_SmallAreaLowGrayLevelEmphasis + 0.49760476 * wavelet-LHH_glcm_SumSquares −0.20140729 * original_glszm_LowGrayLevelZoneEmphasis − 0.48808121 * wavelet-HHH_glszm_SmallAreaEmphasis − 0.29281344 * wavelet-LLH_glcm_ClusterShade − 0.20921238 * wavelet-HHL_glcm_Imc2. The scatter plots of the radiomics score for each patient in the training and validation sets are shown in **Figure S1**.

### Diagnostic Validation of Radiomics Signature

Radiomics score of LNM-positive group was significantly higher than that of LNM-negative group in the training set (median, −0.238 *vs* −0.918; p < 0.001), and the difference was then confirmed in the validation set (median, −0.420 *vs* −1.137; p = 0.017). The AUC values of radiomics signature were 0.733 [95% confidence interval (CI): 0.623, 0.843) in the training set and 0.721 (95% CI: 0.550, 0.892) in the validation set, indicating good predictive efficacy.

### Development, Performance, and Validation of the Radiomics Nomogram

**Figure 4:**
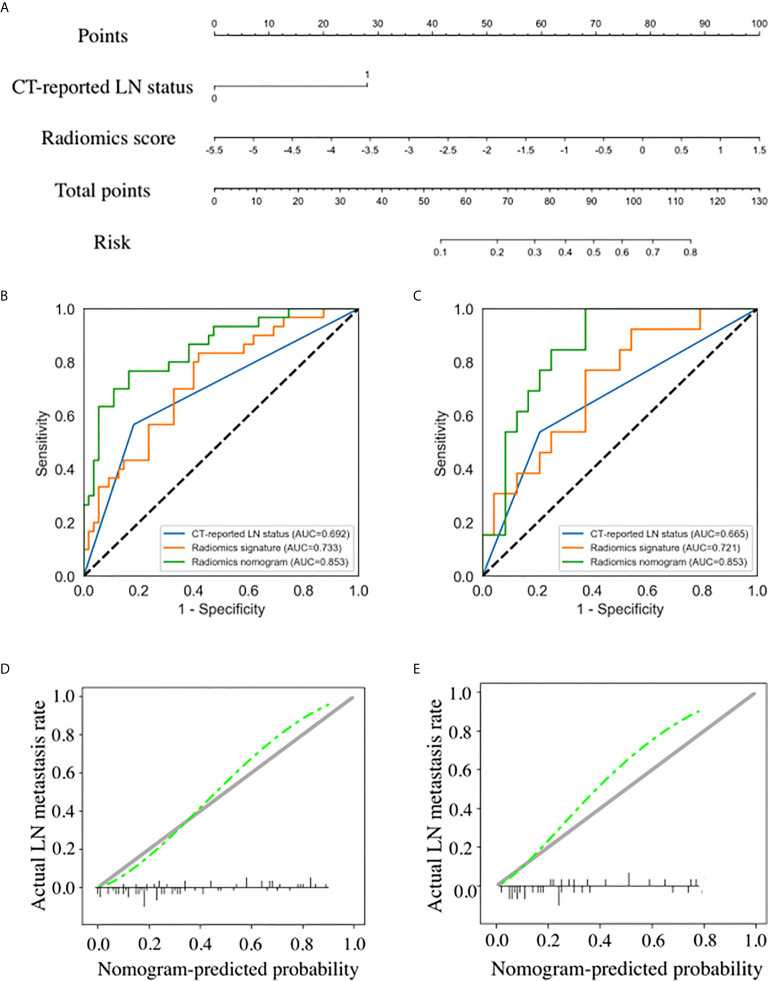
Radiomics nomogram developed with receiver operating characteristic (ROC) curves and calibration curves. **(A)** A radiomics nomogram incorporating the radiomics signature and CT-reported lymph node (LN) status was developed in the training set. Comparison of ROC curves between the CT-reported LN status, radiomics signature, and radiomics nomogram for the prediction of LN metastasis in the training set **(B)** and validation set **(C)**. Calibration curves of the radiomics nomogram in the training set **(D)** and validation set **(E)**.

Results of the univariate and multivariate logistic regression analyses are listed in **Table 3**. The VIFs of the five potential predictors ranged from 1.09 to 2.43 in collinearity diagnosis, indicating that there was no collinearity in these factors (**Table S2**). In the multivariate logistic regression analysis, radiomics signature and CT-reported LN status were identified as independent predictors of LN metastasis in PC (**Table S3**). A radiomics nomogram that incorporated these two predictors was constructed. All ROC curves are shown in **Figure 4**. In the training set, the radiomics nomogram demonstrated the highest discrimination capability, with the AUC of 0.853 (95% CI: 0.767, 0.939), which was higher than the radiomics signature [AUC, 0.733 (95% CI: 0.623, 0.843); p = 0.004] and CT-reported LN status alone [AUC, 0.692 (95% CI: 0.589, 0.796); p = 0.001]. In the validation set, the radiomics nomogram provided the greatest AUC (0.853; 95% CI: 0.731, 0.975), which indicated that the radiomics nomogram achieved the best predictive efficacy than the radiomics signature [AUC, 0.721 (95% CI: 0.550, 0.892); p = 0.064] and CT-reported LN status alone [AUC, 0.665 (95% CI: 0.501, 0.829); p = 0.016]. The specific performances of the models are summarized in **Table 4**.

**Table 3:** Risk factors for LN metastasis in periampullary carcinomas.

Variable	Univariate logistic regression		Multivariate logistic regression	
	Odds ratio	P value	Odds ratio	P-value
Gender	0.96 (0.36, 2.53)	0.930	NA	NA
Age	1.15 (0.58, 2.27)	0.695	NA	NA
Tumor size	4.08 (1.31, 12.74)	0.015	4.99 (0.89, 27.98)	0.068
CT-reported LN status	5.88 (2.17, 15.92)	0.001	6.53 (2.04, 20.88)	0.002
CT-reported vascular invasion	2.67 (1.05, 6.76)	0.039	0.30 (0.05, 1.67)	0.167
Tumor origin	6.13 (1.84, 20.46)	0.003	4.02 (0.79, 20.57)	0.095
CA 19-9	0.94 (0.34, 2.58)	0.903	NA	NA
CA 125	3.86 (0.34, 44.41)	0.279	NA	NA
CEA	1.89 (0.25, 14.17)	0.534	NA	NA
Radiomics score	3.27 (1.57, 6.81)	0.002	2.60 (1.04, 6.49)	0.041

Data in parentheses are 95% confidence intervals. CA 125, carbohydrate antigen 125; CA 19-9, carbohydrate antigen 19-9; CEA, carcinoembryonic antigen; LN, lymph node; NA, not available. These variables were eliminated in the multivariate logistic regression model, so the odds ratio and p-values were not available.

**Table 4:** Performances of the CT-reported LN status, radiomics signature, and radiomics nomogram.

Model	Training set (n = 85)				Validation set (n = 37)			
	Accuracy	AUC (95% CI)	Sensitivity	Specificity	Accuracy	AUC (95% CI)	Sensitivity	Specificity
CT-reported LN status	72.94%	0.692 (0.589, 0.796)	56.67%	81.82%	70.27%	0.665 (0.501, 0.829)	53.85%	79.17%
Radiomics signature	67.06%	0.733 (0.623, 0.843)	83.33%	58.18%	67.57%	0.721 (0.550, 0.892)	76.92%	62.50%
Radiomics nomogram	81.18%	0.853 (0.767, 0.939)	76.67%	83.64%	78.38%	0.853 (0.731, 0.975)	61.54%	87.50%

AUC, area under the curve; CI, confidence interval; LN, lymph node.

Nomogram score of LNM-positive group was significantly higher than that of LNM-negative group in the training set (median, 0.613 *vs* 0.186; p < 0.001), and the difference was then confirmed in the validation set (median, 0.423 *vs* 0.156; p = 0.001). The calibration curves of the radiomics nomogram showed good consistency between predicted LNM probability and actual LNM rate in both training and validation sets. For the training set, a non-significant statistic (p = 0.689) of the H–L test suggested no significant deviation from an ideal fitting. The favorable calibration of the radiomics nomogram was further confirmed in the validation set. For the validation set, the H–L test yielded a p-value of 0.278 (**Figure 4**).

### Clinical Utility of the Radiomics Nomogram

**Figure 5:**
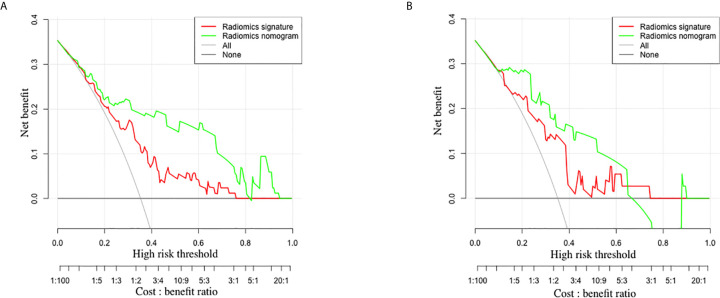
The decision curves of radiomics signature and radiomics nomogram in the training set **(A)** and validation set **(B)**. The y-axis indicates the net benefit. The x-axis indicates the threshold probability at a range of 0.0 to 1.0. The red and green dotted lines represent the decision curves of radiomics signature and radiomics nomogram, respectively. The light gray line represents the decision curve of the assumption that all patients suffer from LN metastasis, and the dark gray line represents the decision curve of the assumption that no patients suffer from LN metastasis. The radiomics nomogram had higher net benefit than radiomics signature.

The decision curve analysis for the radiomics nomogram and the radiomics signature are presented in **Figure 5**. In both training and validation sets, the radiomics nomogram showed a larger area under the decision curves, indicating that compared with “treat-all” or “treat-none” strategies, the radiomics nomogram adds more net benefit to predict LN metastasis for threshold probabilities of more than 11%.

## Discussion

In this study, a radiomics nomogram for non-invasive preoperative prediction of LN metastasis in patients with PC was developed and validated. The nomogram was constructed by incorporating the radiomics signature and CT-reported LN status and demonstrated favorable discriminative ability in both the training set (AUC, 0.853) and the validation set (AUC, 0.853), outperforming conventional morphology-based diagnostic criteria for LN staging on CT images.

PCs remain a diagnostic and therapeutic challenge. The potentially curative option for patients with PC is pancreaticoduodenectomy. However, by the time of presentation, nearly 70–80% of PCs are unresectable due to lymph node involvement, invasion of adherent organs or vessels, and distant metastasis (26, 27). It is worth noting that patients with para-aortal LN metastases should be considered contraindicated for resection since positive para-aortal LNs were reported to be associated with poor survival after pancreatoduodenectomy (13, 28). In addition to traditional surgical methods, neoadjuvant therapy has been investigated in patients with PC. Previous studies revealed that through the fibrosis of LNs, the lymph node ratio is reduced, and patients with preoperative LNM could benefit from a better prognosis (10, 29). Furthermore, LN metastasis has been suggested as an important predictor of patient prognosis. Nappo et al. found that compared with patients with LNM, the median overall survival of patients without LNM was significantly longer (32 *vs* 69 months, respectively; p < 0.05) (30). Therefore, accurate preoperative assessment of LN metastasis is crucial for optimal treatment planning and prognosis prediction.

In daily clinical practice, image-based differentiation of metastatic LNs from non-metastatic LNs mainly depends on morphological features and sizes of LNs, but subjectivity exists in this procedure. Moreover, metastatic LNs with small sizes and benign LNs with non-specific inflammatory hyperplasia are also inevitable. Therefore, correct diagnosis of LN metastasis using conventional imaging modalities is difficult for radiologists. A systematic review and meta-analysis carried out by Tseng et al. revealed that conventional CT imaging has a low diagnostic accuracy (63–81%) in assessing LN metastasis in PC (13), which was verified in our study. Fine-needle aspiration has been regarded as a valuable method for the diagnosis of LN metastasis, but it is invasive, and its ability to detect small metastatic LNs is limited (17).

Alternatively, radiomics could overcome the above-mentioned shortcomings. Radiomics enables the non-invasive detection of the underlying relationship between invisible quantitative image features and pathophysiological characteristics. With the rapid development in the radiomics research field, more than 1,000 radiomics features are now available for more comprehensive presentation of tumors (31). In this study, seven texture features related to tumor heterogeneity were selected to build the radiomics signature, which were supposed to reveal tumor characteristics hidden behind the speckle (32). Histogram-based features depend on a single pixel value, which are calculated based on the overall intensity distribution. On the other hand, texture features are calculated based on the local distribution of voxel. Texture features consider the interaction between neighboring pixels and are therefore more suitable for quantifying tumor heterogeneity (33). This is consistent with previous studies that the efficacy of gray level co-occurrence matrix (GLCM)/gray level size zone matrix (GLSZM)-based texture features for capturing heterogeneous texture information is better than that of histogram-based features (34). In addition, we used VOI for feature selection in our study. Compared with 2-D ROI which was drawn on the largest cross-sectional slice, this 3-D ROI could reflect the heterogeneity of the whole tumor volume, reduce the omission of certain important features on a single slice, and achieve an improvement in the discrimination efficacy (31, 35).

In the manual tumor segmentation procedure, the reproducibility of radiomics features is the most important and unsettled aspect, which would be affected by the subjectivity when determining the tumor boundary. To ensure that the selected features are robust and reproducible, we used the ICCs to evaluate the interobserver reliability and intraobserver reproducibility, and only features of both kinds of ICC with values larger than 0.90 were selected for the subsequent analysis. In our study, LASSO algorithm was used for feature redundancy elimination. This method has two main advantages. First, it allows features to be selected based on their univariable association with the outcome without overfitting. Second, it enables a signature to be constructed by a panel of selected covariates (17, 36). In addition, we used one-way ANOVA between the above-mentioned two steps to select the most LN metastasis-related features to construct the predictive model. Our radiomics signature comprised of seven robust features and indicated good predictive efficacy.

In our study, we developed a radiomics nomogram as an individualized tool for prediction of LN metastasis in PCs and evaluated whether decisions based on the nomogram could benefit patients. Decision curve analysis was used to assess the clinical consequences based on threshold probability, from which a net benefit could be derived (37–39). Our results documented that, given a threshold probability of more than 11%, the radiomics nomogram-based LNM detection approach outperformed either the treat-all or treat-none scheme in both training and validation sets. Notably, the presented radiomics nomogram consists of only two items that are easily accessible from routine CT images. Thus, the nomogram developed from our current study can be used as a reliable and non-invasive modality to preoperatively predict LN metastasis in PCs.

A couple of limitations of our study should be acknowledged. First, due to the retrospective nature of our study, selection bias was difficult to avoid. Patients with an advanced tumor stage and incompetent for surgery were excluded. Second, the sample size was limited to this single-center study. Prospective multi-center study of this rare tumor is needed to obtain external validation from other hospitals in future. Third, the tumor segmentation was manually performed by the radiologists, which was time-consuming and labor-intensive. Further computer algorithm-assisted automatic segmentation should be used. Fourth, the possibility of micrometastasis in LNM-negative cases cannot be excluded. However, the clinical significance of LN micrometastasis remains controversial. Finally, genomic characteristics were not incorporated in this study. As a newly emerging field in oncology, radiogenomics integrates radiomic characteristics with genomic phenotypes and could reflect underlying gene expression or mutation status. Further studies focusing on this topic should be proposed.

In conclusion, we proposed a convenient and non-invasive radiomics nomogram that incorporates radiomics signature and CT-reported LN status to preoperatively predict LN status in patients with PC in order to facilitate clinical decision making and predict patient survival after surgery.

## Data Availability Statement

The original contributions presented in the study are included in the article/**Supplementary Material**. Further inquiries can be directed to the corresponding author.

## Ethics Statement

The studies involving human participants were reviewed and approved by Ethics Committee of Shandong Provincial Hospital Affiliated to Shandong First Medical University. Written informed consent for participation was not required for this study in accordance with the national legislation and the institutional requirements.

## Author Contributions

LB, JX, and ZH contributed to conception and design of the study. LB, TZ, KL, and MD contributed to collecting and analyzing the CT images. JX and CH performed the radiomics aspects and statistical analysis. LB, YL, and JX wrote the draft of the manuscript. JX, XW, CH, XM, and ZH contributed to revision of the manuscript. All authors contributed to the article and approved the submitted version.

## Funding

This work was supported by a grant from the Taishan Scholars Project (XW), the Natural Science Foundation of Shandong (NO. ZR2020MH289), and Academic Promotion Program of Shandong First Medical University (2019QL023).

## Conflict of Interest

Authors JX and CH are employed by Beijing Deepwise & League of PHD Technology Co., Ltd.

The remaining authors declare that the research was conducted in the absence of any commercial or financial relationships that could be construed as a potential conflict of interest.

## Publisher’s Note

All claims expressed in this article are solely those of the authors and do not necessarily represent those of their affiliated organizations, or those of the publisher, the editors and the reviewers. Any product that may be evaluated in this article, or claim that may be made by its manufacturer, is not guaranteed or endorsed by the publisher.
