# Supplementary material for: A CT-Based Radiomics Nomogram for Preoperative Prediction of Lymph Node Metastasis in Periampullary Carcinomas
Source: Front Oncol. 2021 Jul 29;11:632176. doi: 10.3389/fonc.2021.632176 (PMC8358686; doi:10.3389/fonc.2021.632176)
Supplement: Supplementary file 1 [file DataSheet_1.docx]

**Supplementary Materials**

**Radiomic Feature**

In our study, seven texture features (including five wavelet-based features and two original features) were selected to build the radiomics signature, which were based on gray level co-occurrence matrix (GLCM) /gray level size zone matrix (GLSZM).

1. GLSZM

Let:

- N_g_ be the number of discreet intensity values in the image
- N_s_ be the number of discreet zone sizes in the image
- N_p_ be the number of voxels in the image
- Nz be the number of zones in the ROI, which is equal to
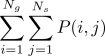
 and
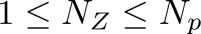

-
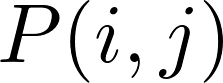
 be the size zone matrix.
-
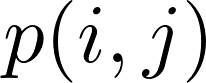
 be the normalized size zone matrix, define as
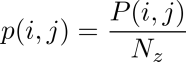


wavelet-HHH_glszm_SmallAreaEmphasis


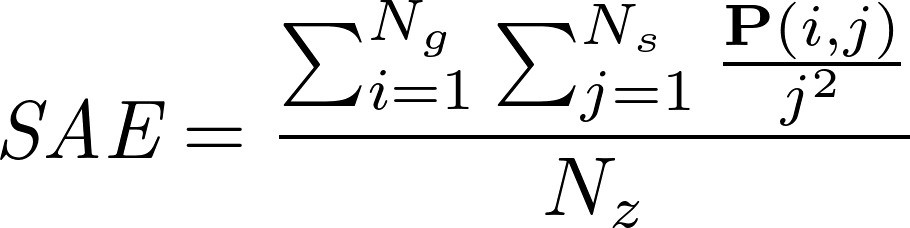


original_glszm_LowGrayLevelZoneEmphasis


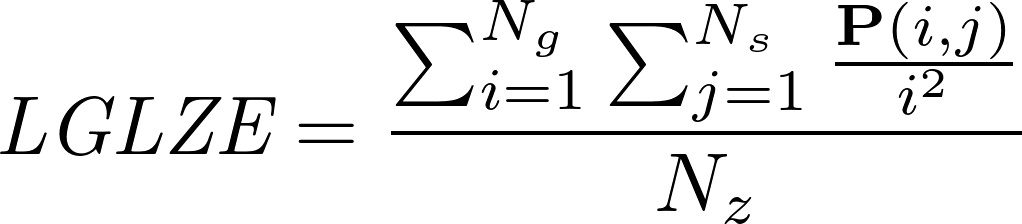


original_glszm_SmallAreaLowGrayLevelEmphasis


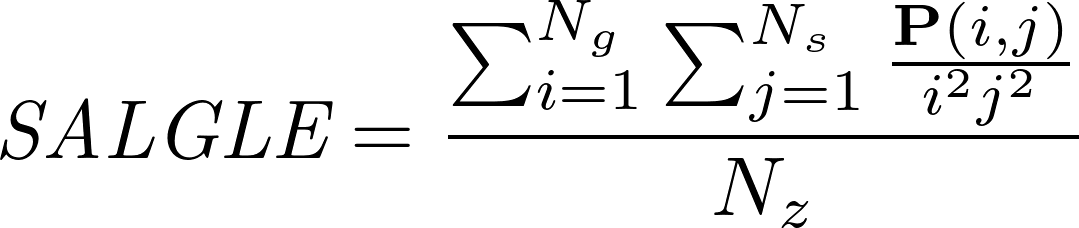


wavelet-LLH_glszm_GrayLevelNonUniformity


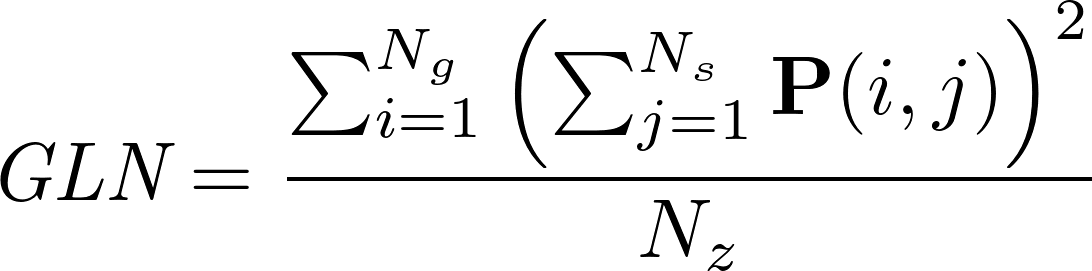


1. GLCM

Let:

-
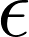
 be an arbitrary small positive number (2.2x10^-16^)
-
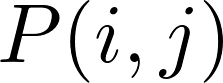
 be the co-occurence matrix for an arbitary
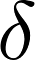
 and
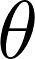

-
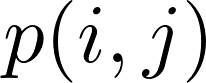
 be the normalized co-occurence matrix and equal to
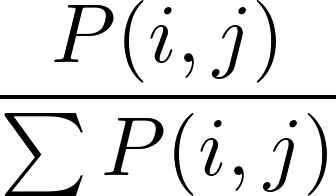

- N_g_ be the number of discrete intensity levels in the image
-
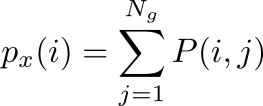
 be the marginal row probabilities
-
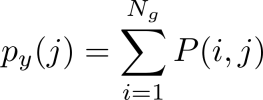
be the marginal column probabilities
-
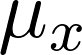
 be the mean gray level intensity of
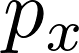
 and defined as
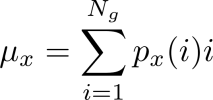

-
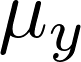
 be the mean gray level intensity of
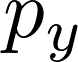
 and defined as
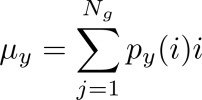

-
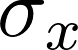
 be the standard deviation of
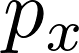

-
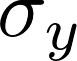
 be the standard deviation of
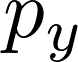

-
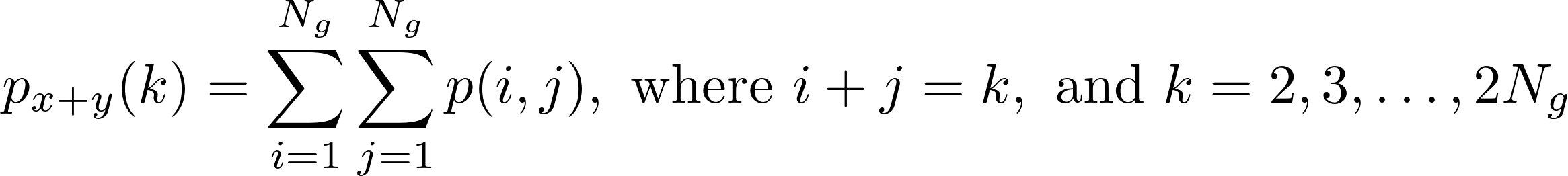

-
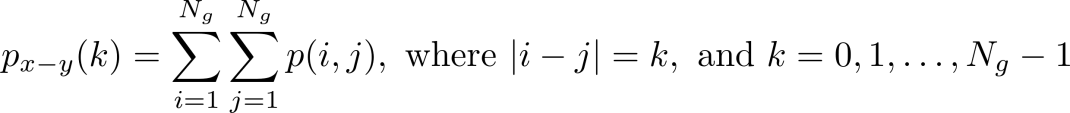

-
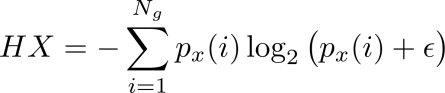
 be the entropy of
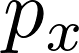

-
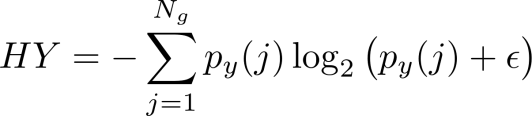
 be the entropy of
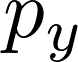

-
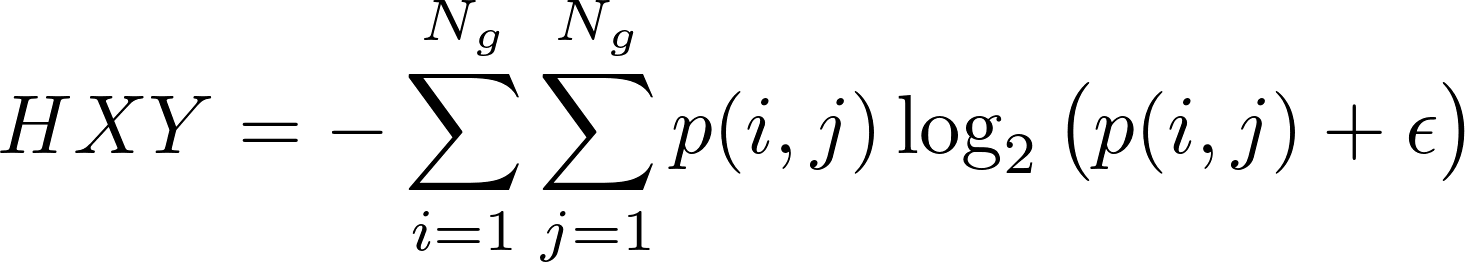
 be the entropy of
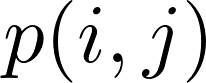

-
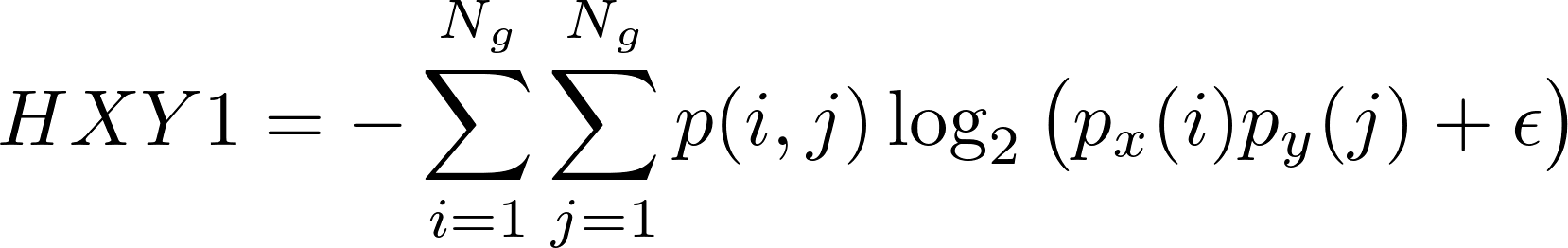

-
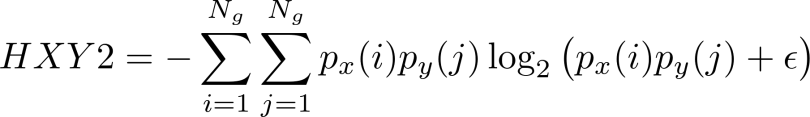


wavelet-LLH_glcm_ClusterShade


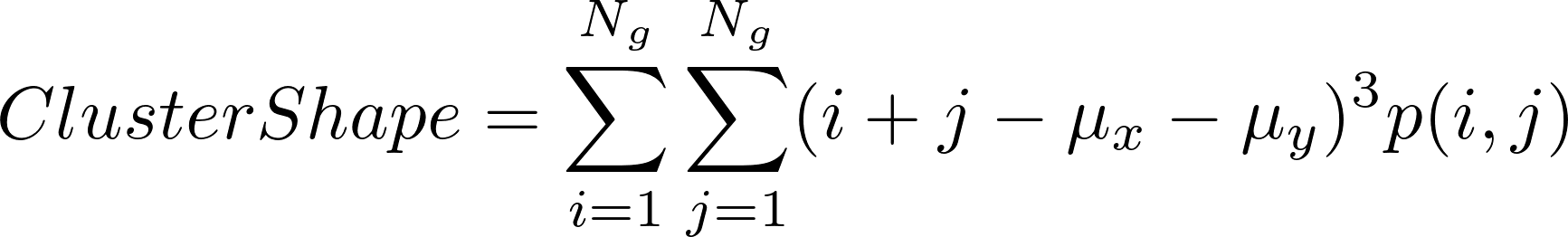


wavelet-HHL_glcm_Imc2


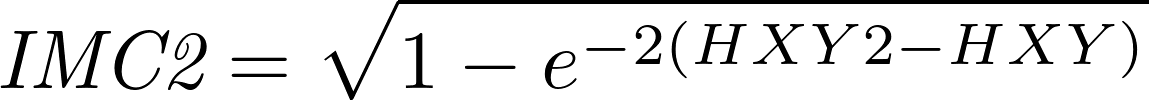


wavelet-LHH_glcm_SumSquares


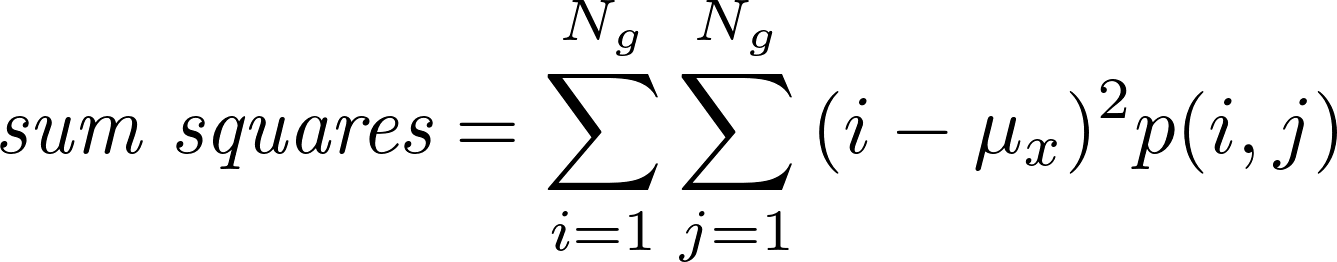


**Table S1 Baseline characteristics of the training set and validation set**

| Characteristic | Training set  (n = 85) | Validation set  (n = 37) | P value |
| --- | --- | --- | --- |
| Age (mean ± SD) | 57.72 ± 11.90 | 60.49 ± 10.67 | 0.207 |
| Gender |  |  | 0.320 |
| Male | 60 (70.6) | 22 (59.5) |  |
| Female | 25 (29.4) | 15 (40.5) |  |
| Tumor size (> 3 cm) | 16 (18.8) | 7 (18.9) | 0.811 |
| CT-reported LN status |  |  | 0.892 |
| LN negative | 58 (68.2) | 25 (67.6) |  |
| LN positive | 27 (31.8) | 12 (32.4) |  |
| CT-reported vascular  invasion | 30 (35.3) | 13 (35.1) | 0.850 |
| Tumor origin |  |  | 0.854 |
| Duodenum | 24 (28.2) | 10 (27.1) |  |
| Ampulla of Vater | 16 (18.9) | 8 (21.6) |  |
| Common bile duct | 12 (14.1) | 7 (18.9) |  |
| Pancreas | 33 (38.8) | 12 (32.4) |  |
| CA 19-9 (> 39 U/ml) | 63 (72.1) | 26 (70.3) | 0.218 |
| CA 125 (> 39 U/ml) | 3 (3.5) | 2 (5.4) | 0.987 |
| CEA (> 10 ng/ml) | 4 (4.7) | 4 (10.8) | 0.393 |
| Radiomics score (mean ± SD) | -0.84 ± 1.34 | -0.97 ± 1.01 | 0.537 |

Note: Data are number of patients; data in parentheses are percentage unless otherwise indicated. CA 125 = carbohydrate antigen 125, CA 19-9 = carbohydrate antigen 19-9, CEA = carcinoembryonic antigen, LN = lymph node, SD = standard deviation.

**Table S2 VIFs for all the candidate variables in the logistic regression analysis**

| Variable | VIF |
| --- | --- |
| CT-reported LN status | 1.090070 |
| Radiomics score | 1.390886 |
| Tumor origin | 1.504284 |
| Tumor size | 1.702389 |
| CT-reported vascular invasion | 2.432624 |

Note: LN = lymph node, VIF = variance inflation factor.

**Table S3 Multivariable analysis for features used in the nomogram**

| Variable | Coefficient | P value | Odds ratio (95% CI) |
| --- | --- | --- | --- |
| Radiomics score | 0.8586 | 0.0031 | 3.2544 (1.4884, 7.1157) |
| CT-reported LN status | 1.6872 | 0.0020 | 5.4046 (1.8493, 15.7950) |

NOTE: LN = lymph node.


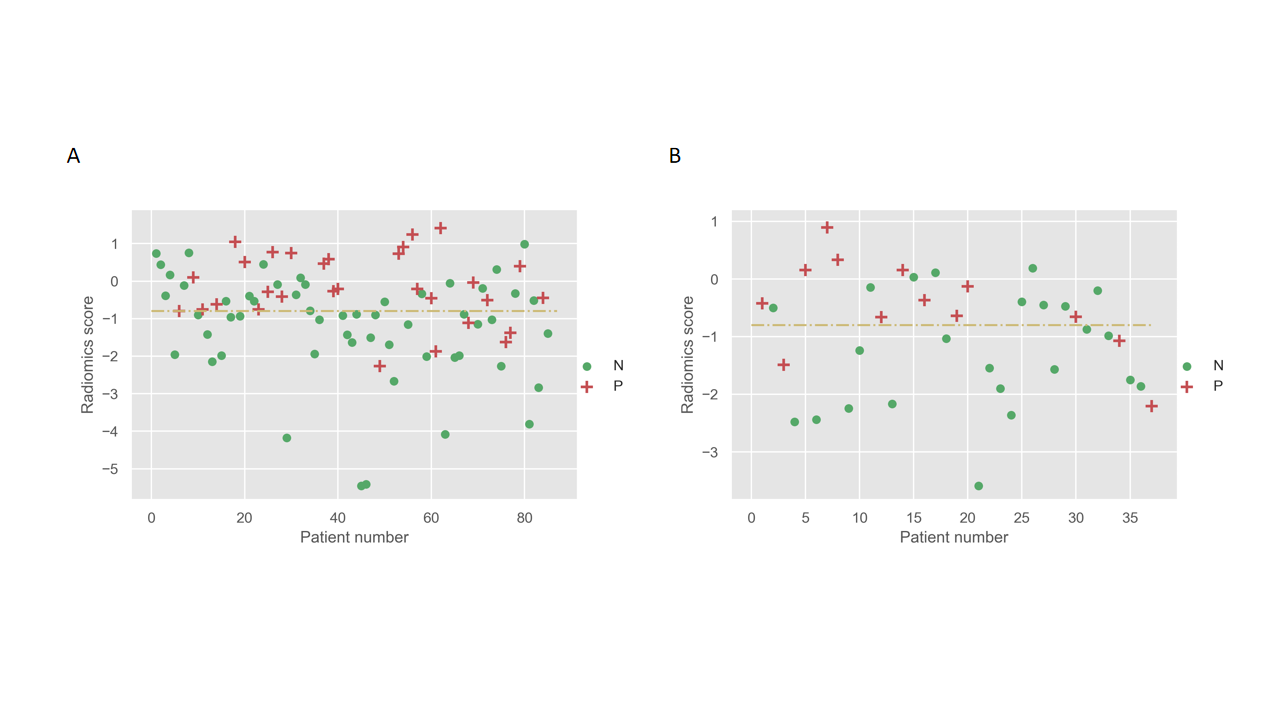
**Figure S1**. The scatter plot of the radiomics score for each patient in training set (A) and validation set (B). The red markers indicate patients with synchronous LN metastasis; the green markers indicate patients without LN metastasis. The yellow horizontal line represents the threshold.
